# Closed Intramedullary Locking Nailing for Metacarpal Fractures: A Retrospective Study of Sixty-Six Fractures

**DOI:** 10.5704/MOJ.1807.002

**Published:** 2018-07

**Authors:** C Ghazala, N Choudhry, A Rajeev

**Affiliations:** Department of Orthopaedics, Gateshead Health NHS Foundation Trust, Gateshead, United Kingdom

**Keywords:** metacarpal fractures, locked intramedullary nailing, complications

## Abstract

**Introduction:** Metacarpal fractures are frequent injuries in the young male working population and the majority are treated non-operatively. There is a growing trend to surgically treat these fractures, with the aim of reducing the deformity and shortening the rehabilitation period. The aim of this retrospective case series is to report on our experience and clinical outcomes of using percutaneous flexible locking nails for the management of displaced metacarpal fractures. This study is a retrospective review of 66 fractures that were managed at our centre over a 7-year period.

**Materials and Methods:** Records of 60 patients were retrospectively reviewed. Indications for surgery were a displaced metacarpal shaft or neck fracture with associated rotational deformity, or multiple metacarpal fractures. The fracture was reduced by closed manipulation, and a flexible pre-bent locked intramedullary nail (1.6mm diameter) was inserted through a percutaneous dorsal antegrade approach, facilitated by a specially designed pre-fabricated awl. The implant was removed at union. Patients were followed-up in clinic until the fracture had united.

**Results:** The mean union time was seven weeks (range 2 to 22 weeks) and there were nine (14%) delayed unions (>3 months) and no non-unions. The nail had migrated in three cases (5%) and caused skin impingement in two cases (3%). There was one infected case (2%). Rotational clinical deformity was evident for two (3%) cases.

**Conclusion:** The use of a minimally-invasive locked intramedullary nailing for unstable metacarpal fractures has a significantly low complication rate, with predictable union times and good functional outcomes.

## Introduction

The incidence of metacarpal fractures is greatest in the young male population, where these injuries are often sustained during an assault (predominately affecting the fifth ray) or sporting activity^1^. Metacarpal fractures most frequently involve the neck and the management of metacarpal fractures varies considerably^2^.

Surgical intervention is indicated when there is rotational deformity, an open fracture, a fracture involving multiple metacarpals, evidence of displaced intra-articular involvement, or when an acceptable reduction cannot be achieved conservatively^2^. Nevertheless, fifth metacarpal neck fractures with volar angulation of up to 70 degrees have had satisfactory functional outcomes without formal reduction, while up to 10 degrees angulation can be accepted for the second and third rays^3-4^. The normal neck-shaft angle of 15 degrees should be considered when reviewing radiographs^5^.

Operative treatment for metacarpal fractures includes percutaneous Kirschner (K-wire) wire or dorsal plate stabilisation for neck and shaft fractures^2^. Interfragmentary screw compression is a further option for shaft fractures, though this procedure is technically challenging and is best suited for long oblique fractures. Intramedullary K-wire stabilisation of extra-articular metacarpal neck and shaft fractures has the advantage of being minimally invasive and avoids disrupting the extensor tendon; however, this technique rarely achieves absolute stability^2,5^.

Gonzalez and co-workers reported a technique for the internal stabilisation of metacarpal fractures using pre-bent flexible intramedullary nails^6^. This technique inserted up to four 0.8mm pre-bent rods proximal or distal to the fracture site with few complications, and since these rods were pre-bent, it was possible to achieve rotational control of the distal fragment. Orbay and colleagues developed this further, enhancing the stabilisation via a proximal locking pin, thus achieving 3-point fixation, minimising the risks of axial shortening and rotation and permitting this method of stabilisation for the more unstable comminuted and spiral fracture configurations^7-8^.

The aim of this retrospective case series was to report on our surgical experience of managing metacarpal fractures using flexible and pre-bent locking intramedullary nails. This procedure was indicated for any significantly displaced or unstable metacarpal fracture, or clinically rotated digit where non-operative treatment was inappropriate. Contraindications towards this management were intra-articular fractures and significant diaphyseal comminution.

## Materials and Methods

This is a retrospective case series, covering a seven-year period, of patients who sustained metacarpal fractures and were surgically managed at our centre using 1.6 or 1.1mm diameter locked metacarpal nails [small bone fixation system, Hand Innovations, Miami, FL]. The procedures were performed by our consultant orthopaedic surgeons, and/or an associate specialist surgeon or intern (junior) surgeon under appropriate supervision. There were 374 surgically-treated metacarpal fractures during the study period. The inclusion criteria were all the patients with metacarpal fractures who were treated with locked metacarpal nails. The exclusion criteria were proximal metacarpal fractures, intra-articular fractures and thumb metacarpal fractures.

A computerised database was used to generate a list of patients who were treated with this implant between April 2007 and August 2014. Electronic and conventional medical records, including radiographs, were reviewed and the following data items were recorded for each patient: gender; age at diagnosis; hand dominance; occupation; mechanism of injury; fracture location and classification; approximate union time and lastly, complications.

Finally, each patient from this cohort was sent by post a structured questionnaire (Quick-DASH) with the aim of assessing his/her post-operative function; we also made telephone calls to those patients who did not respond to the questionnaire to help improve our response rates^9^. The scores were converted to a percentage of functional disability, where 0% is good function and 100% is poor function.

The general operative technique was as follows. Following induction of general or regional anaesthesia, the patient was transferred to a laminar-flow operation theatre and positioned supine with the shoulder abducted to 90 degrees on an arm board. Fluoroscopic guidance was used during the procedure. An above-elbow tourniquet was applied and inflated to 250mmHg. Standard skin preparation and draping was performed prior to tourniquet inflation.

**Fig. 1:**
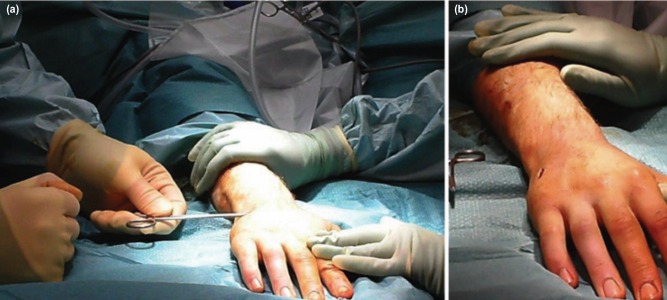
(a) Fluoroscopic guidance is used to confirm reduction by closed manipulation and the entry point is located at the base of the metacarpal. (b) A small stab incision is made over the entry point and the extensor tendon is retracted. Dissection is performed to expose the dorsal cortex.

**Fig. 2:**
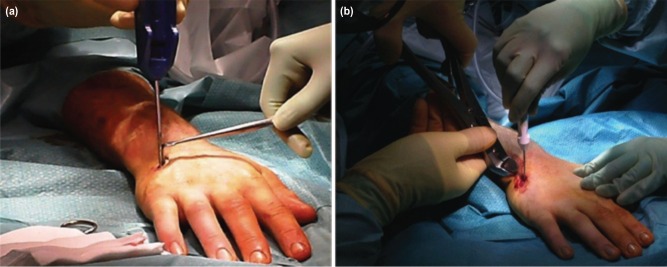
(a) The awl is gently advanced into the dorsal cortex and the metacarpal nail is passed via the awl into the medullary canal, up to the fracture site. Following manipulation and radiological confirmation of reduction, the nail is advanced beyond the fracture site to the subchondral bone. (b) Proximal locking is achieved by introducing the cannulated pin into the end of the bent nail and advancing it into the metacarpal base.

Closed reduction of the fractured metacarpal is achieved and confirmed using fluoroscopy; the entry point at the metacarpal base is also located (Fig. 1a). A small stab incision is made over the entry point, with soft tissue dissection down to the dorsal cortex; care is taken to retract the extensor tendon away from the field (Fig. 1b). The awl is advanced into the dorsal cortex and the nail is passed via this device into the medullary canal, up to the fracture site (Fig. 2a). Fluoroscopy is used at this stage to confirm adequate reduction of the fracture, and the nail is then advanced into the distal fragment to the subchondral bone of the metacarpal head; the proximal end of the nail is then cut from the handle using a pair of wire cutters. Proximal locking improves longitudinal and rotational stability and this can be achieved via the proximal locking pin. The proximal end of the cut nail is bent approximately 90 degrees to the shaft using the cannulated wire bender, and the cannulated locking pin is passed via the end of the nail and advanced into the metacarpal base (Fig. 2b). Finally, the protruding ends of the nail-pin construct are cut so that they are deep to the skin, and the soft tissue protection cap is applied to this free end. The skin is closed in layers, covering the implants. The hand is immobilised in a modified volar Edinburgh plaster slab for one week for soft tissue healing. The patient is instructed to begin immediate mobilisation of the interphalangeal joints, and reviewed in clinic in one week and the dressing is removed. At this stage, the patient is transferred to a futuro splint. The rehabilitation after this period is specific to the patient but will usually consist of physiotherapy. The patient is reviewed at various intervals over approximately eight weeks until the fracture has united, at which stage elective removal of the implant is planned.

## Results

Sixty patients (Table I) were identified (54 males, 6 females); the median age was 23 years (range: 15 to 69 years). Patients generally had manual labour occupations (43%, 26 patients). The majority (62%, 37 patients) of fractures were the result of a direct blow to the metacarpal; falls were the next most common mechanism of injury (18%, 11 patients). The dominant hand was involved in 52 cases (87%) and the little finger was the most common metacarpal to be fractured (62%, 37 patients); this was followed by the ring finger metacarpal in 13 cases (22%).

**Table I:** Patient profiles

Population attributes	Value
Number of patients (treated fractures)	60 (66)
Males (%)	54 (90)
Females (%)	6 (10)
Median age (range)	23 (15-69)
Dominant hand (%)	52 (87)
Mechanism of injury	
Direct blow (%)	37 (62)
Fall (%)	11 (18)
Others (%)	12 (20)

Overall, 66 fractures were treated through locked intramedullary nailing, with six (10%) patients having two adjacent metacarpal fractures nailed (Table II). Of the 66 fractures nailed, fractures involving the metacarpal shaft predominated (65%, 43 fractures; mean angulation 38 degrees). In 23 (35%) patients there was a metacarpal neck fracture (mean angulation 53 degrees). Overall, a transverse fracture pattern was present in half of all fractures nailed; whereas, an oblique configuration was noted in just under a quarter (16 fractures; 24%).

**Table II:** Fracture attributes

Fracture attributes	Value
Total number of fractures nailed	66
Little finger metacarpal nailing (patients)	37
Ring finger metacarpal nailing (patients)	13
Ring and little finger metacarpal nailing (patients)	6; 12 fractures
Index metacarpal nailing (patients)	3
Middle metacarpal nailing (patients)	1
Metacarpal shaft fractures (mean angulation)	43; 65% (38^0^)
Metacarpal neck fractures (mean angulation)	23; 35% (53^0^)
Transverse (%)	33 (50)
Oblique (%)	16 (24)
Spiral (%)	8 (12)
Comminuted (%)	9 (14)

Overall mean union time was seven weeks (range 2 to 22 weeks) and there were nine (14%) delayed unions (>3 months) and no non-unions (Table III). The nail had migrated in three cases (5%); one case was due to noncompliance of the patient where he removed his own cast. The nail caused skin impingement for two cases (3%). There was one infected case (2%). Of the 66 fractures nailed, clinical deformity was evident in five (8%) cases, while three (5%) had signs of a depressed knuckle and two (3%) had minimal clinical rotational deformity at follow-up. No patients had tendon irritation.

**Table III:** Surgical outcomes and complication rates

Surgical outcomes and complications	Value
Mean union time (weeks)	7; range 2 - 22
Delayed unions (%)	9 (14)
Non-unions	0
Nail migration (%)	3 (5)
Rotational deformity (%)	2 (3)
Skin impingement (%)	2 (3)
Infected cases (%)	1 (2)

The one non-compliant patient (unemployed male; 22 years; punch injury involving the index finger of the dominant hand), had undergone intramedullary nailing for a comminuted metacarpal head fracture, but he had removed his cast and the nail had displaced, thus necessitating revision surgery with a further metacarpal nail. Clinical deformity was evident on final follow-up with a shortened metacarpal and bony lump on the dorsal aspect of the hand.

The functional outcome study was conducted using the Quick-DASH questionnaire and patients were contacted to complete the questionnaire via postal response and telephone interviews. The response rate was 24% (n=16), and these patients were more than two years following their metacarpal surgery (range 2 to 7 years). The mean calculated Quick-DASH score was 0.51 (range 0 to 2.64), or 13% functional disability (range 0% to 57% of functional disability). Fifty-six percent (n=9) of responding patients had reported no signs of functional disability (Quick-DASH score of 0).

## Discussion

**Fig. 3:**
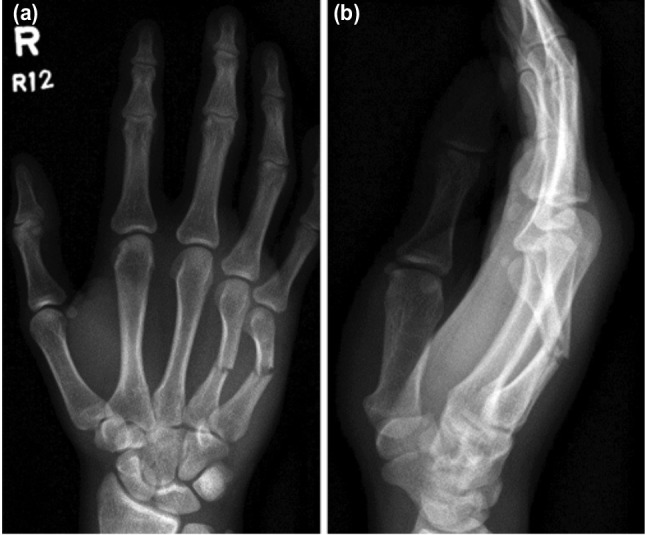
Pre-operative radiographs of (a) PA and (b) Lateral views of transverse mid metacarpal ring and little finger shaft fractures.

Metacarpal fractures are the most frequent type of hand injury; however, the majority are managed non-surgically. Surgery is indicated for cases with rotational deformity, multiple shaft fractures (Fig. 3), intra-articular involvement and significant displacement^10-11^.

**Fig. 4:**
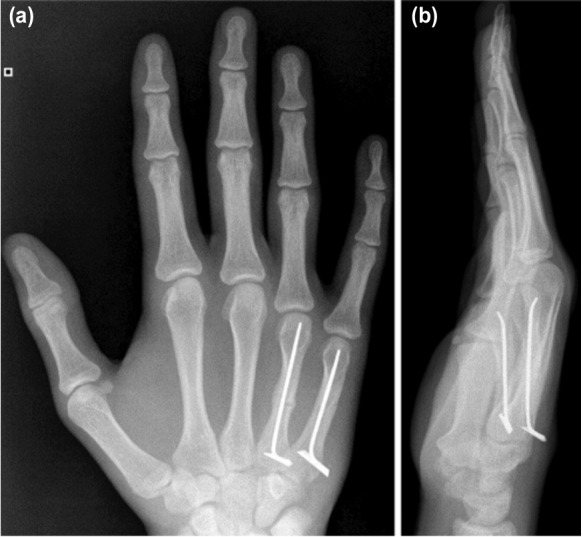
Post-operative radiographs (a) PA and (b) Lateral views of united transverse mid metacarpal ring and little finger shaft fractures with the locked intramedullary nail in-situ.

Many surgical techniques have been described and these include intraosseous wiring, percutaneous K-wires, crossed K-wires, intra-medullary K-wires, flexible intramedullary nails and the traditional plate and screw fixation ^6,7,12-19^. Our centre has demonstrated that locked intramedullary metacarpal nailing achieves an appropriate fracture stabilisation and union rate with relatively few complications, and that complications are largely influenced by patient compliance (Fig. 4). Our results complement findings from other studies where flexible intramedullary nailing has been performed for unstable metacarpal fractures (Table IV).

**Table IV:** Comparative studies of metacarpal fracture stabilisation

Authors	Surgical intervention	Year	Outcome measures	Author conclusion
Orbay *et al*^21^	Locked and non-locked flexible nail	2006	Radiograph measures, mean operating time, ROM, grip strength, VAS for pain,	Metacarpal shaft fractures treated with IM nail achieve good functional outcomes with low complication rates
Ozer *et al*^22^	IM nail vs plate screw fixation	2008	complication rates ROM, DASH and radiological evaluation	IM nails do not provide better outcomes to plate screw fixation. Although shorter operative times in IM nail vs plate screw fixation. An increased rate of complications in IM nailing noted in patients with fractured distal third metacarpal
Venkatachalam *et al*^17^	Locked flexible IM nail	2011	Clinical & radiographic evaluation, ROM, complication rates	Locked flexible IM nailing can be used in unstable metacarpal fracture with good functional outcomes and low complication rates
Fujitani e*t al*^19^	Low profile plates vs intramedullary nail	2012	Radiographic measure, Grip strength, post-operative complication rates	Patients that prefer minimally invasive surgery or good ROM should have IM nailing, whereas patients requiring early recovery of strong hand function should have plate fixation
Boussakri et al^18^	Pre-bent elastic IM nail	2014	Clinical & radiological evaluation, ROM	Recommends IM nailing in all metacarpal fractures
Ghazala *et al* (this study)	Locked flexible IM nail	2016	Clinical evaluation, DASH and complication rates	Appropriately selected patients have a good functional outcome with low complication rate

Prompted by the wide array of fixation techniques for unstable metacarpal fractures and no consensus on definitive management, a systematic review was performed by Corkum *et al*^20^. Interestingly, this group reported that all intramedullary fixation techniques for metacarpal fractures appeared to have equivalent or better outcomes to other surgical techniques. Other reported benefits of intramedullary nailing have been demonstrated by studies from both Orbay and Ozer who reported that percutaneous intramedullary fixation is technically quicker to perform compared to alternative methods of stabilisation^21,22^. Moreover, Orbay and Touhami reported that nailing could also be used in complex fracture patterns such as spiral or comminuted metacarpal fractures with high patient satisfaction and good functional outcomes^21^. In their series of 95 metacarpal fractures treated with a locking intramedullary device, two patients each had tendon irritation and penetration of nail into the MCP joint and incidence of delayed union. This is in contrast to our study in which 14% had delayed union and 5% nail migration, but no cases of tendon irritation. Patients who have had intramedullary nailing have also demonstrated an improved range of finger motion in comparison to traditional plate screw fixation^19^. Fujitani *et al* reported that compared to plate fixation, intramedullary nailing had a lower risk of soft tissue compromise and periosteal stripping at the fracture site^19^. This resulted in minimal adherent scar formation around the extensor mechanism and metacarpophalangeal joint.

Despite the advantages, a strong consensus remains between investigators who conclude plate-screw fixation provides the most definitive type of stabilisation in metacarpal fractures in comparison to all other methods including intramedullary nailing^23-25^. This appears to be in concordance with findings by Curtis *et al* who performed a biomechanical comparative analysis between the intramedullary nail, crossed K-wire and plate screw constructs using composite bones^25^. Their data demonstrated that the average load to failure was significantly greater in plate-screw fixation compared to the other two techniques. Furthermore, plate fixation was around 11-15 times as stable as intramedullary nailing or crossed K-wires, respectively.

Most recently Zhang and colleagues reported that plate-screw fixation in the longer term required a significantly longer rehabilitation, when compared to intramedullary nails; however, early rehabilitation in plate-screw fixation allowed patients to return to their manual job earlier compared to patients with intramedullary nails^26^. The earlier return of hand function in terms of grip strength is also supported by Fujitani *et al*, although this outcome has not been reported in other earlier case series^19^.

Other groups also report a greater incidence of complications with intramedullary nailing compared to plate-screw fixation^22,26^. The most frequently reported complications were the loss of reduction, nail migration and tendon irritation requiring removal of metacarpal plates. Page and Stern in their study of 63 metacarpal fractures treated with plates and screws reported a non-union rate of 1.6%, delayed union rate (4.8%), plate problems including plate prominence, loosening or breakage (6.3%), infection (3.1%) and tendon rupture 1.6%^27^. Fusetti *et al* reviewed the results of 157 metacarpals treated with plates and screws and observed a non-union rate of 15%, stiffness (10%), plate loosening or breakage (8%), complex regional pain syndrome (two patients), and one patient who developed a deep infection^28^. Nevertheless, these studies had no statistical significance in clinical and patient outcomes between intramedullary nailing and conventional plate screw fixation.

Our study has several limitations. Radiographic measurements were performed by a single clinician. Our results would be improved by calculating the interobserver error. Despite our best efforts, functional follow-up using the Quick-DASH survey provided us with a response rate of 24%. Secondly, a further limitation was that this was a retrospective study of medical records and quantitative functional assessment was attempted by later posting the Quick-DASH questionnaire to patients. Lastly, there was no control comparison to other cheaper fixation modalities.

## Conclusion

For displaced metacarpal neck or shaft fractures, locked intramedullary metacarpal nailing is a technically simple method of maintaining fracture stability following reduction, with good functional outcomes.

## Conflict Of Interest

None of the authors have any conflict of interest in this study. This manuscript has not been submitted for consideration elsewhere and has not been previously published. There are no financial interests associated with this study, including the authors and institution involved.
